# Agreement and Sensitivity of the Acceleration–Velocity Profile Derived via Local Positioning System

**DOI:** 10.3390/s24196192

**Published:** 2024-09-25

**Authors:** Mladen Jovanović, Adriano Arguedas-Soley, Dimitrije Cabarkapa, Håkan Andersson, Dóra Nagy, Nenad Trunić, Vladimir Banković, Répási Richárd, Sandor Safar, Laszlo Ratgeber

**Affiliations:** 1 Faculty of Sport and Physical Education, University of Belgrade, 11000 Belgrade, Serbia; 2 School of Health Sciences, Western Sydney University, Sydney, NSW 2751, Australia; 3 High Performance Department, Greater Western Sydney Giants Football Club, Sydney, NSW 2751, Australia; 4 Jayhawk Athletic Performance Laboratory—Wu Tsai Human Performance Alliance, Department of Health, Sport and Exercise Sciences, University of Kansas, Lawrence, KS 66045, USA; 5 High Performance Center, 35246 Vaxjo, Sweden; 6 Faculty of Health Sciences, Doctoral School of Health Sciences, University of Pecs, 7621 Pecs, Hungary; 7 Faculty of Health Sciences, Institute of Physiotherapy and Sport Science, University of Pecs, 7621 Pecs, Hungary; 8 Faculty of Physical Culture and Sports Management, Singidunum University, 11000 Belgrade, Serbia; 9 Center for Basketball Methodology and Education, 7621 Pecs, Hungary; 10 Institute of Sport, Training Theory and Methodology Research Center, University of Physical Education, 1123 Budapest, Hungary; 11 Department of Sports Games, Institute of Sport, University of Physical Education, 1123 Budapest, Hungary

**Keywords:** sport, athlete, sprinting, testing, performance, GPS, LPS, speed, in situ

## Abstract

Sprint performance is commonly assessed via discrete sprint tests and analyzed through kinematic estimates modeled using a mono-exponential equation, including estimated maximal sprinting speed (MSS), relative acceleration (TAU), maximum acceleration (MAC), and relative propulsive maximal power (PMAX). The acceleration–velocity profile (AVP) provides a simple summary of short sprint performance using two parameters: MSS and MAC, which are useful for simplifying descriptions of sprint performance, comparison between athletes and groups of athletes, and estimating changes in performance over time or due to training intervention. However, discrete testing poses logistical challenges and defines an athlete’s AVP exclusively from the performance achieved in an isolated testing environment. Recently, an in situ AVP (velocity–acceleration method) was proposed to estimate kinematic parameters from velocity and acceleration data obtained via global or local positioning systems (GPS/LPS) over multiple training sessions, plausibly improving the time efficiency of sprint monitoring and increasing the sample size that defines the athlete’s AVP. However, the validity and sensitivity of estimates derived from the velocity–acceleration method in relation to changes in criterion scores remain elusive. To assess the concurrent validity and sensitivity of kinematic measures from the velocity–acceleration method, 31 elite youth basketball athletes (23 males and 8 females) completed two maximal effort 30 m sprint trials. Performance was simultaneously measured by a laser gun and an LPS (Kinexon), with kinematic parameters estimated using the time–velocity and velocity–acceleration methods. Agreement (%Bias) between laser gun and LPS-derived estimates was within the practically significant magnitude (±5%), while confidence intervals for the percentage mean absolute difference (%MAD) overlapped practical significance for TAU, MAC, and PMAX using the velocity–acceleration method. Only the MSS parameter showed a sensitivity ($\%MDC_{95}$) within practical significance (<5%), with all other parameters showing unsatisfactory sensitivity (>10%) for both the time–velocity and velocity–acceleration methods. Thus, sports practitioners may be confident in the concurrent validity and sensitivity of MSS estimates derived in situ using the velocity–acceleration method, while caution should be applied when using this method to infer an athlete’s maximal acceleration capabilities.

## 1. Introduction

The ability to produce and sustain high speed and acceleration represents a key physical performance determinant in team sports such as football, rugby, and basketball. Maximum velocity and acceleration are routinely assessed via short sprints in many team sport settings to inform athlete preparation, monitoring, and rehabilitation strategies [1,2]. Assessing sprint performance characteristics using laser gun devices, which record instantaneous velocity–time traces, is common practice in sports settings. The velocity–time relationship can then be modeled using a mono-exponential equation [3] to describe an athlete’s acceleration–velocity profile (AVP), yielding estimations of sprint mechanical characteristics such as maximal sprinting speed (MSS), relative acceleration (TAU), maximal acceleration (MAC), slope of the acceleration speed ($AS_{slope})$, and maximum net relative propulsive power ($P_{max}$) [2,4,5]. Accordingly, it has been proposed that by quantifying individual differences and training-induced changes in sprint mechanical characteristics, practitioners can better understand the factors limiting an athlete’s sprint performance [6].

The development of global positioning systems (GPS), local positioning systems (LPS), and video-tracking systems (VID) in team sports has enabled the estimation of velocity and acceleration outputs within the training and competition environment [7]. These systems provide an attractive, time-efficient alternative to applied practitioners, which could allow for monitoring of sprint performance without having to schedule discrete testing of full squads or expose athletes to additional maximal sprint efforts. Indeed, sprint time data derived from LPS have demonstrated high test-to-test reliability (intra-class correlation coefficient (ICC): 0.99–1.00) and concurrent validity (mean absolute error (MAE): 0.01–0.02) with single-beam timing gate data in ice-hockey athletes [8]. However, LPS use has also been reported to incur greater measurement error (4%) for velocity measures during sport-specific tasks (e.g., small-sided games in football) compared to GPS (2.2%) and VID (2.7%) technology [9], with error magnitudes for all three systems increasing at higher velocities. Nonetheless, it was recently reported in football and handball athletes performing small-sided games that errors for instantaneous peak velocity derived from LPS (percentage deviation: 0.7–1.7%) were smaller than or consistent with documented errors for comparable systems [10], suggesting that LPS technology provides an adequate means of monitoring positional and velocity-related outputs of team sport athletes.

Monitoring the AVP of an athlete is of particular interest to performance practitioners, as it represents a simple model to estimate the kinematics of an athlete’s short sprint performance. Recently, an embedded (i.e., in situ) AVP has been proposed using GPS or LPS data collected over multiple training sessions [8,10,11,12], possibly enabling the assessment of individual sprint-acceleration abilities within the athlete’s sport-specific environment and eliminating the need for discrete sprint testing. Moreover, outcome measures from individual in situ AVPs have been used to differentiate sprint mechanical properties between youth footballers of differing maturity status [13] and have demonstrated stable measurement properties in elite football athletes (standard error of measurement (SEM): MSS: 0.41, MAC: 0.36, $AS_{slope}$: 0.07) [12]. Certainly, quantification of an athlete’s AVP during sport-specific activities, namely their acceleration capacity at different velocities, could provide valuable information for sport performance practitioners to individualize training prescriptions, monitor fitness–fatigue states, or profile talented youth athletes [6,13,14]. However, ensuring the appropriate validity and sensitivity of existing technologies is essential in athlete monitoring, particularly for quantifying parameters from short-duration sprints [15] derived from GPS or LPS systems, which inherently sample at lower frequencies (10–100 Hz) than criterion velocity measurements (e.g., laser guns, 2.56 KHz). To date, the measurement properties of LPS-derived AVP in team sports remain unexplored.

Therefore, this study aimed to evaluate the concurrent validity of LPS-derived sprint mechanical outcome measures (MSS, TAU, MAC, and $P_{max}$) using the embedded AVP previously detailed in the literature [12,16] by quantifying the agreement with data sampled simultaneously from a reference standard laser gun during short sprints performed by youth basketball athletes. Further, the sensitivity of the LPS-derived outcome measures in detecting changes in the criterion (laser gun) scores was also examined.

## 2. Materials and Methods

### 2.1. Participants

This study involved the participation of 31 basketball players, comprising 23 males (age = 16.1 ± 1.0 years; height = 188.3 ± 7.5 cm; body mass = 69.5 ± 10.8 kg) and 8 females (age = 16.1 ± 1.4 years; height = 170.5 ± 7.5 cm; body mass = 60.9 ± 7.6 kg). All athletes were selected from the highest youth level in Hungary. The participants were duly apprised of the potential hazards and advantages of their involvement in the study, and a written authorization was procured from both the participants and their parents. The research adhered to the University of Belgrade ethical guidelines and was conducted in accordance with the most recent version of the Declaration of Helsinki.

### 2.2. Procedures

Before evaluating sprint performance, a standardized warm-up protocol lasting 15 min was executed. The warm-up protocol involved a series of mobility and low-intensity running exercises performed repeatedly within a 20 m distance, culminating in three incremental sub-maximal sprints covering a distance of 30 m. Following the warm-up procedure, the participants executed two trials of maximal sprints covering a distance of 30 m, with a minimum rest period of 3 min between each trial. Participants were given a “set” command to take and maintain a stationary position, after which the laser gun was initiated. After a few moments, an additional “go” signal was given, with athletes initiating sprinting when they felt ready. If equipment failure occurred, an additional sprint was executed as needed. This was necessary for another research interest involving photocells [2]. Due to this strategy, one to three sprints were collected and measured for every participant. The velocity measurements were continuously recorded for each attempt utilizing a laser gun (CMP3 Distance Sensor, Noptel Oy, Oulu, Finland) at a sampling rate of 2.56 KHz. A polynomial function was used to model the relationship between distance and time, which was subsequently resampled at a frequency of 1000 Hz through the use of Musclelab™ v10.232.107.5298 (Ergotest Technology AS, Langesund, Norway). The laser gun was positioned roughly 3 m from the initial timing gate, while the reference point (i.e., zero distance) was established at 1 m behind the initial timing gate. All of the sprints were executed within the confines of an indoor basketball facility.

Positional data were continuously captured with an LPS (20 Hz, Kinexon GmbH, Munich, Germany) throughout all experimental trials. The LPS setup incorporated a specific local network access, 1 power-over-ethernet switch, 1 server, and 26 hardwired anchors secured ∼8 m above the court’s surface. LPS devices were securely positioned between each athlete’s scapulae. The devices communicated via ultra-wideband technology with the anchors to achieve real-time localization and were equipped with an inertial measurement unit (IMU: accelerometer, gyroscope, and magnetometer). The Kinexon LPS has demonstrated reliability and validity in measuring instantaneous velocity and acceleration over a range of starting velocities (coefficient of variation (CV) < 10%, ICC > 0.9; mean biases: velocity < 0.5 km/h, acceleration < 0.01 $ms^{-2}$*)* [17]. Raw positional data were then exported using manufacturer software (basketball-specific app; version 10-3-9) and processed using the R language [18].

To compare the short-sprint mechanical parameters, (1) the maximal sprinting speed (MSS), expressed in meters per second ($ms^{-1}$); (2) the relative acceleration (TAU), expressed in seconds (s); (3) the maximal acceleration (MAC), expressed in meters per second squared ($ms^{-2}$); and (4) the net relative propulsive power ($P_{max}$), expressed in Watts per kilogram (W/kg), were estimated by using the open-source {shorts} package [19,20] available in the R language [18].

**Figure 1:**
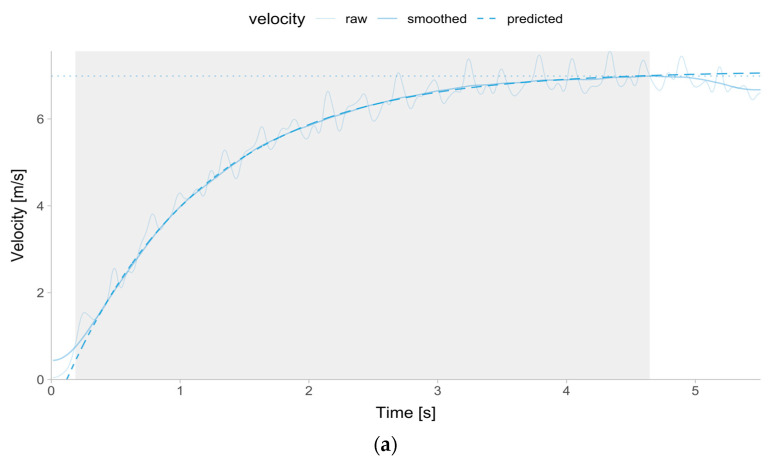

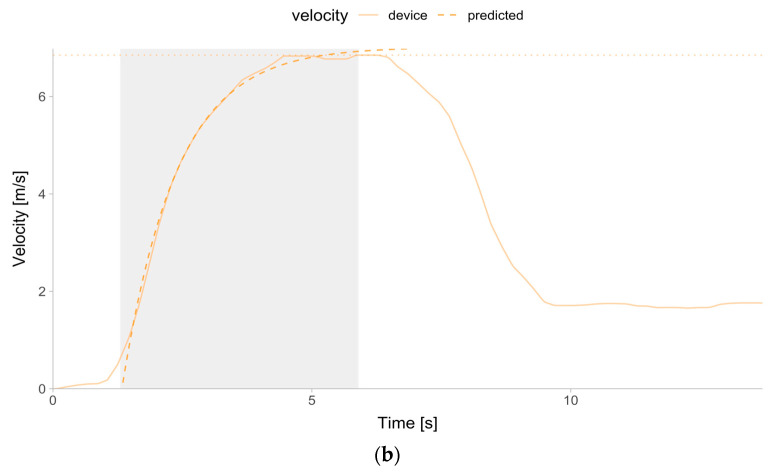
The grey rectangle represents time–velocity data that are used to train the model (Equation (1)), which involves velocity over 0.75 $ms^{-1}$ until the observed peak velocity is reached (indicated by a dotted horizontal line). (**a**) Laser Gun. The thin solid line indicates raw velocity (sampled at 1000 Hz). The thick solid line indicates smoothed velocity (the exact filtering/smoothing method is a proprietary secret of Ergotest Technology AS). The thick dashed line represents the mono-exponential model prediction. (**b**) Kinexon. The thick solid line indicates the reported device velocity (sampled at 20 Hz). The thick dashed line represents the mono-exponential model prediction.

Sprint mechanical parameters for the laser gun were estimated using the smoothed (the exact filtering/smoothing method is a proprietary secret of Ergotest Technology) velocity–time signal and time correction mono-exponential model (Equation (1)) [2], after filtering out velocities below 0.75 $ms^{-1}$ and over the peak smoothed velocity provided by the Musclelab™ software (Figure 1a). Additional model observation weighting was utilized by using time (i.e., observations with higher time were given more weight).

(1) $$v\left(t\right)=MSS\times \left(1-e^{-\frac{t+TC}{TAU}}\right)$$

The same sprint parameter estimation procedure was utilized for the Kinexon (Figure 1b). Due to the continuous collection of Kinexon data throughout the whole testing session, specific sprint trials had to be recognized using a bespoke script built in the R language [18] written by one of the authors. For this study, the method of estimating sprint parameters using time–velocity tracing and Equation (1) was named the time–velocity method.

**Figure 2:**
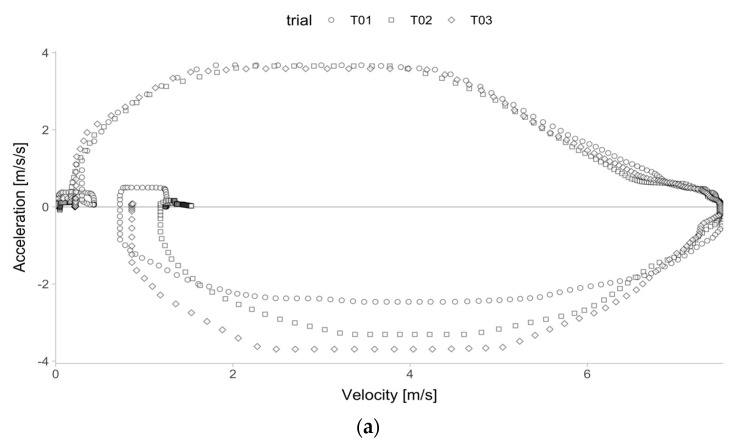

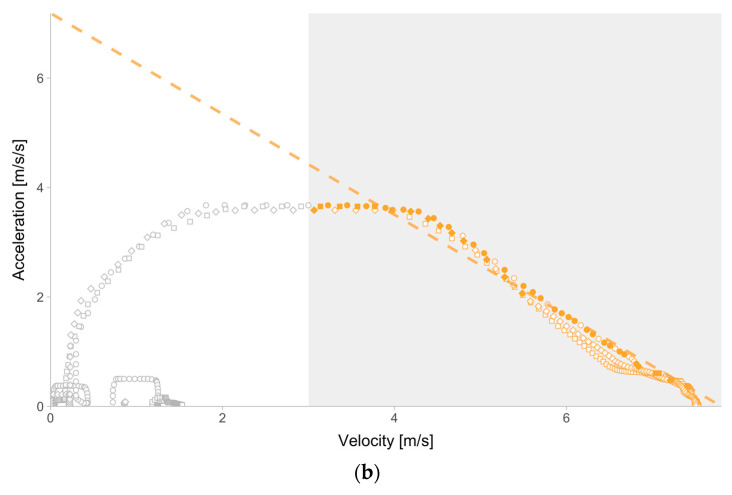
Velocity–acceleration method for estimating MSS and MAC parameters using the Kinexon data for a single individual across three sprint trials. (**a**) Instantaneous velocity and acceleration observations across three sprint trials for a single individual. Note: Trial 1—T01 (○ symbol); Trial 2—T02 (□ symbol); Trial 3—T03 (◇ symbol). (**b**) Using the velocity–acceleration method, only positive acceleration (i.e., over 0 ms^−2^) and velocity over 3 ms^−1^ observations were used (grey rectangle) to estimate MSS and MAC parameters. A linear regression was then fitted (dashed line) using the two maximal acceleration observations that were collected for every 0.2 ms^−1^ increment (filled points). The MAC parameter, which is equivalent to the estimated intercept of the linear regression model, can be visualized as the point where the regression line (dashed line) crosses the y-axis. The MSS parameter, which is equal to the estimated intercept divided by the negative estimated slope of the linear regression model, can be visualized as the point where the regression line (dashed line) crosses the x-axis.

To test the agreement and sensitivity of the embedded (i.e., in situ) sprint profiles [12,16], Kinexon velocity–acceleration data across all trials were used (Figure 2a). This method involves using velocity–acceleration tracing, which was named the velocity–acceleration method for this study. This method is attractive to the practitioners because it does not require any additional manual processing of the continuous monitoring data. The procedure of estimating an acceleration–velocity profile (i.e., MSS and MAC) involves filtering in samples where acceleration is positive (i.e., over 0 $ms^{-2}$) and velocity over 3 $ms^{-1}$, and then computing linear regression from the 2 maximal acceleration points collected for every 0.2 $ms^{-1}$ increment (Figure 2b). Additional model observation weighting was utilized by using velocity (i.e., observations with higher velocity were given more weight).

The velocity–acceleration method for estimating AVP using the Kinexon data utilizes all trials, which can involve 1–3 sprints for which we provide the aggregate or the best profile (i.e., some of the data points in certain velocity zones can be from separate sprints). To provide estimates of the agreement, laser gun estimates using the time–velocity method were aggregated across multiple sprints using the best values for MSS, MAC, TAU, and $P_{max}$. This involves using the maximum estimate for all parameters except the TAU parameter, for which the minimum estimate is utilized.

### 2.3. Statistical Analysis

The agreement between the laser gun and Kinexon for both the time–velocity and velocity–acceleration methods was estimated using the percent difference (%Diff) estimator (Equation (2)), which was calculated for every athlete and trial, as well as the sprint parameter.

(2) $$\%Diff=100\times \frac{\left(Timing\;Gates-Laser\right)}{Laser}$$

In addition to the descriptive analysis of the %Diff involving the mean and 2.5th and 97.5th percentiles (i.e., 95% range), scatterplots with simple linear regression between laser gun and Kinexon estimates were generated. Using individual percent difference scores, percent bias (%Bias, or mean percent difference; Equation (3)) and percent mean absolute difference (%MAD; Equation (4)) were calculated as metrics of agreement between the methods.

(3) $$\%Bias=\frac{1}{N}\sum_{i=1}^{N}\left(\%Diff_{i}\right)$$

(4) $$\%MAD=\frac{1}{N}\sum_{i=1}^{N}\left|\%Diff_{i}-\bar{\%Diff}\right|$$

Practitioners are frequently concerned about whether they may utilize estimated parameter values to monitor changes in the true parameters in addition to estimating agreement between them. Thus, an estimate of the sensitivity represents crucial information to decide whether a given measure can be practically used to monitor changes. A minimal detectable change estimator with 95% confidence ($\%MDC_{95}$) [21,22] was utilized to estimate this sensitivity. The $\%MDC_{95}$ value might be regarded as the minimum amount of change that needs to be observed in the estimated parameter for it to be considered a true change. The sensitivity of the LPS in detecting changes in parameters, estimated using agreement with the laser gun, assumes that there is no random error in laser gun estimates. In other words, this method assumes that the laser gun estimates represent the true parameter values.

The percent residual standard error (%RSE) of the linear regression between laser gun (predictor) and Kinexon (outcome) (Equation (5)) was utilized to calculate $\%MDC_{95}$ (Equation (6)) for short-sprint parameters. Assuming no random error is involved in laser gun estimates, %RSE represents the percent standard error of the measurement (%SEM) in the Kinexon estimates.

(5) $$\%RSE=\sqrt{\frac{\sum_{i=1}^{N}{\left(100\times \frac{y_{i}-\hat{y_{i}}}{\hat{y_{i}}}\right)}^{2}}{N-2}}$$

(6) $$\%MDC_{95}=\%RSE\times \sqrt{2}\times 1.96$$

Statistical inferences for the %Bias, %MAD, and $\%MDC_{95}$ estimators were provided using 5000 bootstrap resamples and 95% bias-corrected and accelerated (BCa) confidence intervals [21,23,24,25] using a custom-written R package by one of the authors [26]. Estimated 95% BCa confidence intervals were used together with a selected 5% practically significant threshold to visually interpret the magnitude inference of the agreement and sensitivity metrics [21,27,28,29,30,31,32,33].

## 3. Results

### 3.1. Data Collected

Overall, the collected sample included 30 athletes and 57 sprint trials, with 1.9 trials per athlete (range: 1 to 3 trials). These sprint trials were treated as independent when estimating the agreement using the time–velocity method. Individual athlete aggregates were used when estimating the agreement using the velocity–acceleration method, resulting in 30 observations.

### 3.2. Descriptive

**Figure 3:**
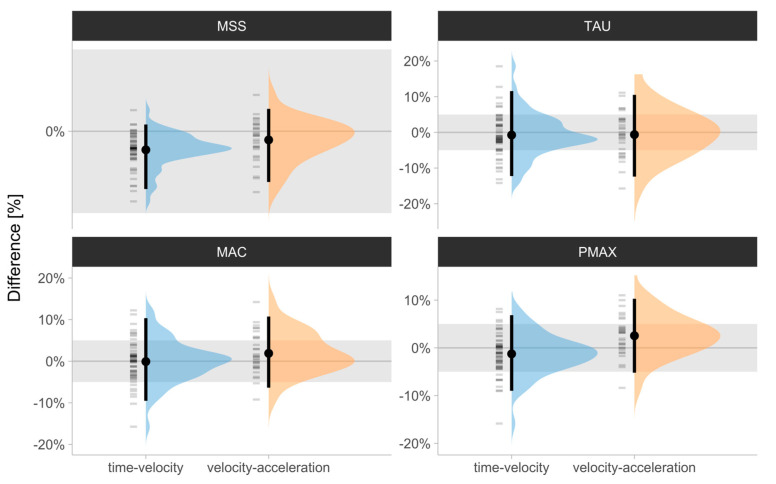
Distributions of the percent difference (%Diff) scores between the laser gun and Kinexon for (1) MSS, (2) TAU, (3) MAC, and (4) $P_{max}$ estimates using (1) time–velocity and (2) velocity–acceleration methods. Error bars represent mean and 2.5th and 97.5th percentiles (i.e., 95% range). Grey bars represent ±5% difference magnitude, used as a visual anchor. MSS—maximum sprinting speed (expressed in $ms^{-1}$); TAU—relative acceleration (s); MAC—maximum acceleration ($ms^{-2}$); $P_{max}$—maximal relative power ($Wkg^{-1}$).

Distributions of the percent difference (%Diff) scores between the laser gun and Kinexon for all parameter estimates using the (1) time–velocity and (2) velocity–acceleration methods, together with the mean and 2.5th and 97.5th percentiles as descriptors, are depicted in Figure 3. Percent differences ranged from −4 to 2.22% for MSS, from −16 to 14.24% for MAC, from −16 to 18.52% for TAU, and from −16 to 11.03% for $P_{max}$ for both the time–velocity and velocity–acceleration methods.

**Figure 4:**
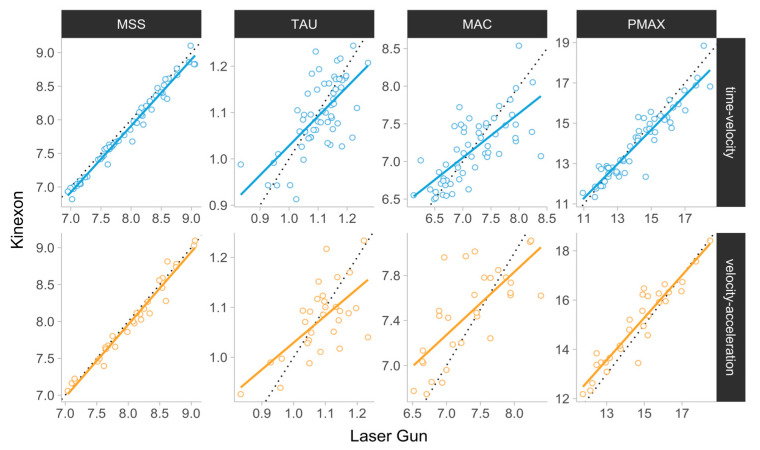
Scatterplot between laser gun and Kinexon estimates for (1) MSS, (2) TAU, (3) MAC, and (4) $P_{max}$ estimates using (1) time–velocity and (2) velocity–acceleration methods. Each circle represents a single athlete trial. The dotted diagonal line represents the identity line, at which all the observations would be positioned for perfect agreement. MSS—maximum sprinting speed ($ms^{-1}$); TAU—relative acceleration (s); MAC—maximum acceleration ($ms^{-2}$); $P_{max}$—maximal relative power ($Wkg^{-1}$).

A scatterplot between the laser gun and Kinexon for all parameter estimates using the (1) time–velocity and (2) velocity–acceleration methods is depicted in Figure 4.

### 3.3. Agreement and Sensitivity

**Figure 5:**
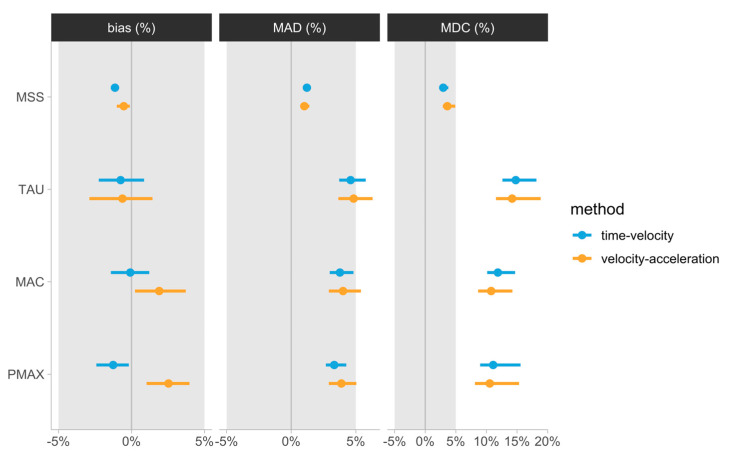
Bootstrapped agreement estimators. Error bars represent 95% bias-corrected and accelerated (BCa) 5000 bootstrap resamples confidence intervals. Grey bars represent ±5% magnitude, used as a visual anchor. MSS—maximum sprinting speed ($ms^{-1}$); TAU—relative acceleration (s); MAC—maximum acceleration ($ms^{-2}$); $P_{max}$—maximal relative power ($Wkg^{-1}$); Bias (%)—mean percent difference; MAD (%)—mean absolute percent difference; MDC (%)—minimum detectable percent difference.

The estimated agreement (%Bias and %MAD) and sensitivity ($\%MDC_{95}$) metrics, together with their 95% BCa confidence intervals and 5% practically significant thresholds, are depicted in Figure 5. Estimated %Bias ranged from −1 to 2.53%, %MAD ranged from 1 to 4.83%, and $\%MDC_{95}$ ranged from 3 to 14.79% across all parameters and for both the time–velocity and velocity–acceleration methods.

## 4. Discussion

Quantifying sprint mechanical characteristics provides valuable insight to practitioners monitoring and profiling athletes involved in sports requiring frequent sprint efforts. The AVP represents a simple, two-parameter model describing the kinematics of an athlete’s short-sprint performance, which may help identify and monitor factors limiting their sprinting abilities [2,16,34]. Traditionally, AVP profiling required athletes to perform maximal sprints during regularly scheduled testing sessions. However, recently, an embedded AVP (velocity–acceleration method) was developed for the estimation of kinematic parameters using GPS or LPS data sampled from training sessions where exposures to maximal accelerations and velocities take place [12,13,34], possibly making sprint monitoring more time-efficient and non-invasive. The present study examined the concurrent validity of LPS-derived (Kinexon) sprint kinematic estimates using the time–velocity and velocity–acceleration methods by assessing their agreement and sensitivity to changes in measures with a reference standard laser gun in elite youth basketball athletes, for the first time, to the best of our knowledge.

The primary finding of the present study was that the agreement between LPS-derived and laser gun estimates (%Bias) fell within the practically significant magnitude of ±5% for all parameters using both the time–velocity and velocity–acceleration methods. Extending on the findings of prior researchers, who have documented good reliability for MSS, MAC, and $AS_{slope}$ using the velocity–acceleration method in team sport athletes (SEM: ~3–8%, ICC > 0.5) [16,34], our results indicate that LPS-derived estimates using the velocity–acceleration method may provide a valid representation of an athlete’s AVP in situ with negligible bias. In addition, these findings are consistent with those reported by Linke et al. [9], showing acceptable accuracy of LPS-derived instantaneous velocity during very-high-speed tasks in football players. However, caution should be applied, as the agreement of TAU, MAC, and $P_{max}$ with laser gun estimates, as determined by %MAD, revealed confidence intervals (CI) crossing the practical threshold of ±5% for both methods (Figure 5). Specifically, using the CI for judgment of statistical significance and the laser gun as the criterion, the velocity–acceleration method showed some misalignment in estimating MAC, TAU, and $P_{max}$, whereas the CI for the time–velocity method only overlapped the practical significance region for TAU. Interestingly, visual inspection of the CIs indicated that apart from $P_{max}$, %Bias values for parameters using the time–velocity and velocity–acceleration methods were not statistically different. Notably, defining the LPS trace using the time-velocity method may be problematic and time-intensive for practitioners, and thus this finding may suggest that the velocity–acceleration (in situ) method can provide an equally valid yet more time-efficient alternative for calculating kinematic estimates besides the $P_{max}$.

Although valid estimation of kinematic estimates is of crucial importance in profiling athletes, the ability to accurately detect changes within and between athletes over time is key in athlete monitoring. To date, the only study to investigate the measurement sensitivity of embedded AVP estimates showed poor internal sensitivity (CV > smallest worthwhile change (SWC)) for GPS-derived MSS, MAC, and $AS_{slope}$ values in elite footballers [12]. In the present study, the sensitivity using $\%MDC_{95}$ was within the practically significant magnitude only for MSS (<5%) using both the time–velocity and velocity–acceleration methods, while the $\%MDC_{95}$ for all other parameters was greater than 10% and up to 15%. This suggests that larger changes in LPS-derived sprint mechanical estimates may be required to ascertain with 95% confidence that a true change in the criterion parameter values (laser gun) took place. Our findings show consistency with a previous investigation from our research group examining the sensitivity of short-sprint mechanical parameters derived from timing gates to changes in laser gun scores [2]. Indeed, the results of this study suggested that the MSS parameter, using the simplest no-correction model, achieved the highest degree of sensitivity ($\%MDC_{95}$ < 7%), and all other parameters demonstrated unsatisfying levels of sensitivity ($\%MDC_{95}$ > 40%) [2]. Interestingly, the $\%MDC_{95}$ values for LPS-derived kinematic estimates in the present study were smaller than those observed for timing gates, potentially offering practitioners a more pragmatic assessment with slightly better sensitivity. Owing to the current findings as well as prior evidence, practitioners may be confident that the MSS indicator has now demonstrated satisfactory sensitivity using both the time–velocity method and velocity–acceleration method. On the other hand, the ability to estimate maximal acceleration traits from available methods remains questionable.

Although our results extend the current understanding of the validity and sensitivity of sprint mechanical parameters derived from novel sprint-profiling methods, this study is not without limitations. Firstly, the embedded AVP (velocity–acceleration method) was originally designed for the assessment of sprint mechanical outcomes during live team sports training and/or match play [12]. In the current study, the embedded AVP was extracted during a discrete sprint-testing session, rather than during a training session with maximal velocity and acceleration efforts, for which the method was intended. However, given that the aim of this study involved assessing the concurrent validity of measures derived from an embedded AVP, the study design necessarily required the inclusion of discrete sprint tests (criterion measure) for comparison of concurrent data samples (i.e., Kinexon-LPS-derived and laser-gun-derived). Although this may mean that the results could be too optimistic compared to when the AVP is eventually estimated during normal training sessions, this study provides a conceptual basis for future applied studies to examine these parameters in situ. Moreover, although we addressed the sensitivity of LPS-derived kinematic estimates using the velocity–acceleration method to changes in criterion scores for the first time to our knowledge, future research should extend these findings by assessing minimum detectable changes over multiple assessments over non-consecutive days, as well as longitudinal training-induced changes in sprint mechanical parameters. Such work could elevate our understanding of whether the embedded AVP is suitable for reliable and valid quantification of positive and negative changes in an athlete’s sprint mechanical characteristics that may subsequently impact their sprint performance.

Furthermore, we hypothesize that the study’s use of an LPS with a 20 Hz sample frequency may have contributed to the TAU, MAC, and $P_{max}$ parameters’ less reliable measurement characteristics. It is recommended that future research investigate the potential effects of raising the LPS sampling frequency on the measurement characteristics of the TAU, MAC, and $P_{max}$ parameters.

## 5. Conclusions

Based on the data obtained in the present investigation, practitioners evaluating LPS-derived estimates of sprint kinematics using the time–velocity and velocity–acceleration methods may have confidence that MSS indices offer satisfactory agreement with and sensitivity to changes in criterion scores. In addition, LPS-derived MSS estimates demonstrated better sensitivity, as per $\%MDC_{95}$, than that reported for timing gates in a similar prior investigation [2]. However, practitioners should also be cautious when using these methods to infer an athlete’s maximum acceleration capabilities, for which the parameters (i.e., TAU, MAC, and $P_{max}$) have shown less robust measurement properties. Future studies should attempt to replicate the current findings and potentially examine longitudinal changes in kinematic parameters using the velocity–acceleration method across both elite and sub-elite team sport athletes.

## Data Availability

The data and the R code can be found at https://osf.io/sk2pa/ (DOI: 10.17605/OSF.IO/SK2PA (16 July 2024)).
